# Oral health of patients with severe rheumatic heart disease

**DOI:** 10.5830/CVJA-2012-009

**Published:** 2012-07

**Authors:** Breminand Maharaj, Ahmed C Vayej

**Affiliations:** Department of Therapeutics and Medicines Management, University of KwaZulu-Natal, Durban, South Africa; Programme: Oral Health, Department of Health, KwaZulu-Natal, Durban, South Africa

**Keywords:** oral health, rheumatic heart disease, periodontal health

## Abstract

**Abstract:**

In order to determine whether adequate attention is paid to the maintenance of good oral health in patients at risk of developing infective endocarditis, we studied 44 black patients with severe rheumatic heart disease before they had cardiac surgery. Plaque and gingival index scores were calculated and panoramic radiographs were done in all patients. There were 17 males and 27 females (mean age: 30.6 years). The plaque and gingival index scores were classified as poor in 31.8 and 54.6% of patients, respectively. Panoramic radiographic findings included caries in 56.8% of patients, peri-apical pathology in 18.1% and retained roots in 22.7% of patients. This study demonstrates that inadequate attention is paid to the maintenance of good oral health in patients with severe rheumatic heart disease. The oral and dental care of patients at risk of developing infective endocarditis needs to be improved.

There are many case reports of the failure of antibiotic prophylaxis to prevent infective endocarditis.^1^ Wahl suggested, however, that ‘failures of prophylaxis’ were actually successful, and that patients had developed endocarditis before or after the dental procedure because of poor oral hygiene.^2^ Individuals who have poor oral health are at high risk of developing spontaneous bacteraemia.^3^

The literature indicates that most cases of infective endocarditis are not related to procedures.^4^,^5^ Eykyn stated that it is becoming increasingly clear that poor dental hygiene rather than dental procedures are responsible for most, if not all, cases of viridans streptococcal endocarditis.^6^ In the study by Pogrel and Welsby, oral sepsis was associated with infective endocarditis in 12% of cases.^7^ Guntheroth pointed out that bacteraemia was found in 11% of patients with oral sepsis and no intervention.^8^

A number of authors have stressed proper oral hygiene as a far more important preventative measure than chemoprophylaxis against infective endocarditis.^2^,^3^,^8^-^11^ In his review of the medical and dental literature on the prevention of infective endocarditis, Barco stated: ‘Finally, and most importantly, dental and periodontal health is the best long-term prevention of infection from the oral cavity for such patients at risk of infective endocarditis’.^12^

Most national guidelines on infective endocarditis prophylaxis stress the maintenance of optimum oral health. The 1997 recommendations of the American Heart Association stated that individuals who are at risk of developing infective endocarditis should establish and maintain the best possible oral health to reduce potential sources of bacterial seeding. They also stated that optimal oral health is maintained through regular professional care.^13^

The Endocarditis Working Party of the British Society for Antimicrobial Chemotherapy has emphasised the need for regular dental attendance for the management of dental health in patients susceptible to infective endocarditis.^14^,^15^ More recent guidelines have stressed the importance of this aspect of prophylaxis.^16^,^17^

The aim of this study was to determine by clinical and radiological examination whether adequate attention is paid to the maintenance of good oral health in black patients who are at risk of developing infective endocarditis.

## Methods

Black patients who had been evaluated by a cardiologist at the cardiac unit at the Wentworth Hospital, Durban, and assessed as having severe rheumatic heart disease requiring valvular surgery, were studied. Patients were examined during the week preceding their cardiac surgery in the cardio-thoracic unit of this hospital after informed consent had been obtained. The study was approved by the Ethics committee of the Nelson R Mandela School of Medicine, University of Natal, Durban.

For each patient, the age, gender and nature of the valvular abnormality were recorded. Plaque and gingival index scores were calculated after careful clinical examination, and rotational panoramic radiographs were done in all patients.^18^-^20^ Each radiograph was examined without magnification using a standard viewing box in a horizontal position. Two observers examined all radiographs; differences in interpretation were resolved by consensus.

Recording sheets were compiled and the presence of the following lesions was recorded: caries, missing teeth, impacted teeth, hypercementosis, peri-apical radiolucencies, attrition, fractured teeth, unerupted teeth, and retained roots. Definitions used for all the radiographic lesions are listed in Table 1.

**Table 1.** Definitions Used For Radiographic Lesions^19^-^21^

Missing teeth	All permanent teeth not on the radiograph
Unerupted teeth	Teeth that had not reached the occlusal plane and which were not impeded from doing so
Impacted teeth	Teeth with fully or incompletely formed roots, impeded by hard tissue from reaching their correct relationship to the occlusal plane and surrounding bone
Hypercementosis	Formation of excessive cementum on tooth roots
Peri-apical radiolucency	Radiolucency associated with the apex of a tooth root
Caries	Radiolucent areas in a tooth
Attrition	Wearing away of a tooth where teeth rub together
Tooth fracture	Fracture of the crown and/or root of the tooth
Retained roots	Roots which cannot be restored because of insufficient remaining healthy tooth tissue and/or supporting tissue
Interdental bone loss	Lack of continuity of lamina dura

## Results

A total of 44 black patients with severe rheumatic heart disease were studied. There were 17 males and 27 females, with ages ranging from 13 to 50 years (mean age: 30.6 years). The distribution of valvular lesions is shown in Table 2. The distribution of scores obtained after calculation of the plaque and gingival index scores are shown in Tables 3 and 4.

**Table 2.** Distribution Of Valvular Lesions (*n* = 44)

*Valvular lesions*	*No. of patients*
Isolated mitral stenosis	8
Isolated mitral regurgitation	3
Mitral stenosis plus regurgitation (mixed mitral valve disease)	16
Aortic regurgitation	3
Aortic regurgitation plus aortic stenosis (mixed aortic valve disease)	3
Mixed mitral valve disease plus mixed aortic valve disease	11

**Table 3.** Plaque Index: Distribution Of Scores

	*Patients*		
*Rating*	*Score*	*Number*	*Percent*
Excellent	0	0	0
Good	0.1–0.9	6	13.6
Fair	1.0–1.9	24	54.6
Poor	2.0–3.0	14	31.8

**Table 4.** Gingival Index: Distribution Of Scores

	*Patients*		
*Rating*	*Score*	*Number*	*Percent*
Excellent*	0	0	0
Good	0.1–0.9	7	15.9
Fair	1.0–1.9	13	29.5
Poor	2.0–3.0	24	54.6

*Healthy tissue

**Fig. 1.**
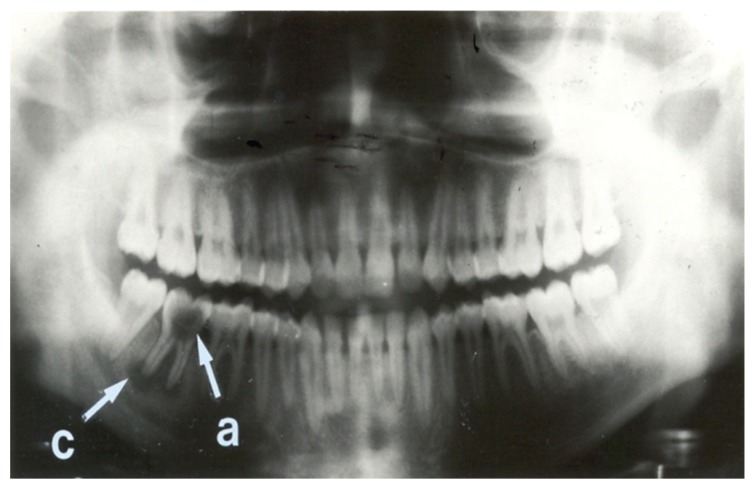
Panoramic radiograph showing few abnormalities. This radiograph shows complete dentition, with no unerupted or impacted teeth. Interdental bone loss is minimal. A large carious lesion (a) is present in the right second mandibular molar tooth. A peri-apical radiolucency (c) is also present at the apex of the same tooth. This is indicative of a peri-apical abscess or granuloma.^21^ The overall condition of the teeth of this patient is good.

**Fig. 2.**
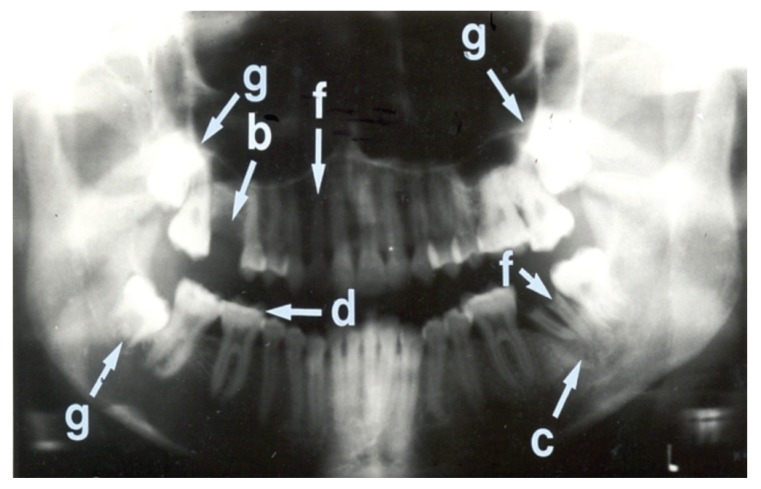
Panoramic radiograph showing more abnormalities than Fig. 1. This radiograph shows three impacted third molar teeth (g). The right first maxillary molar tooth is missing (b). The crown of the left second mandibular molar tooth is missing and retained roots are present (f). The same tooth also shows a peri-apical radiolucency (c) with a well-defined calcified margin indicating possible peri-apical cyst formation.^21^ The retained root of the right second maxillary incisor tooth is also seen (f). The occlusal surfaces of the right mandibular first and second molar teeth show attrition (d).

**Fig. 3.**
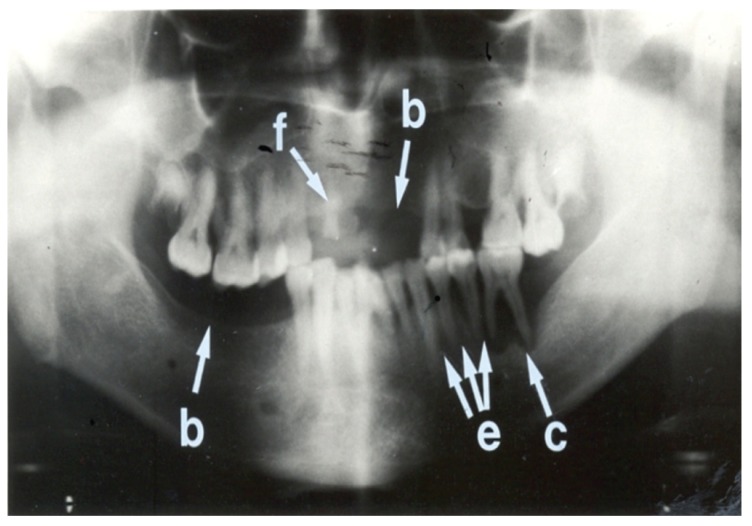
Panoramic radiograph showing more abnormalities than Fig. 2. This radiograph shows edentulous areas (i.e. loss of teeth) involving the right mandibular molar region and the left maxillary incisor and canine regions (b). A retained root of the right maxillary canine tooth is also present (f). Interdental bone loss is seen between the left mandibular premolar and molar teeth (e). A peri-apical radiolucency is associated with the left first mandibular molar tooth (c). The overall condition of the teeth of this patient is poor.

**Fig. 4.**
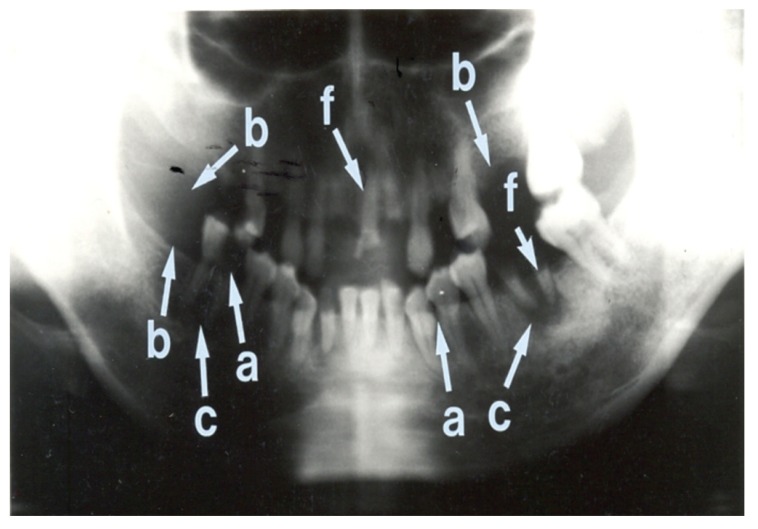
Panoramic radiograph showing many abnormalities. This radiograph shows multiple retained roots (f) and missing teeth (b) in both mandibular and maxillary arches. The right mandibular first molar tooth shows a large carious lesion with loss of crown enamel (a). The left mandibular canine teeth and the left mandibular first premolar tooth show caries interproximally (a). Peri-apical radiolucencies are noted in relation to the right and left mandibular molar teeth (c). The overall condition of the teeth of this patient is extremely poor.

Seven (15.9%) patients had normal panoramic radiographs. No patients were edentulous. The nature of radiographic abnormalities and their relative frequency are shown in Table 5. The panoramic radiographs, shown in Figs 1, 2, 3 and 4, illustrate some of the abnormalities that were detected.

**Table 5.** Types And Frequencies Of Radiographic Abnormalities

	*Patients*	
*Abnormality*	*Number*	*Percent*
Caries	25	56.8
Missing teeth	24	54.5
Peri-apical radiolucencies	8	18.1
Impacted teeth	11	25.0
Unerupted teeth	9	20.5
Hypercementosis	5	11.4
Tooth fracture	1	2.3
Attrition	8	18.2
Retained roots	10	22.7
Interdental bone loss	6	13.6

## Discussion

The plaque and gingival index scores were classified as good in only 13.6 and 15.9%, respectively of patients with severe rheumatic heart disease who were scheduled to have cardiac surgery. None of the patients had a score that would have placed their indices in the excellent category, while 31.8 and 54.6% of patients had gingival and plaque index scores that were rated as poor.

Abnormalities were detected in the panoramic radiographs of 84.1% of patients. The most frequent lesion was caries, present in 56.8% of patients, followed by missing teeth in 54.5%, and impacted teeth in 25% of patients. Retained roots were present in 22.7% and peri-apical pathology was detected in 18.1% of patients. Although seven (15.9%) patients had a normal panoramic radiograph, only one also had both plaque and gingival index scores that were good.

Thom and Howe reported on their study in which 50 patients, all with severe heart disease, were examined clinically and radiologically to ascertain their dental status.^22^ Their study differs from ours in a number of respects. Firstly, they included four patients with congenital heart disease (ventricular septal defect). Secondly, they used full-mouth intra-oral peri-apical radiographs, whereas our study used rotational panoramic radiography.

Thirdly, they decided whether the patients were ‘dentally fit’ and whether they had periodontal disease, although diagnostic criteria were not given. We studied oral health with particular reference to oral hygiene. Fourthly, their study included edentulous patients whereas ours did not have any edentulous patients. Fifthly, their study was done in a developed country.

Nevertheless, comparisons can be made with our study. Furthermore, their findings and conclusions are applicable to developing countries. Thom and Howe reported that 20.5% of patients were dentally fit.^22^ By contrast, only one patient in our group had a normal panoramic radiograph, a plaque index score that was rated as good, and a gingival index score that was good – the latter indicating mild gingival inflammation. Periodontal disease was present in 46% of their patients. Using only the clinical examination, 86.5% of our patients had a fair or poor gingival index. The relative frequency of caries, retained roots and impacted teeth in their study was 20, 34 and 6%, respectively, while in our study it was 56.8, 22.7 and 25%, respectively.

These authors divided their patients into one group (*n* = 11) that visited the dentist regularly (group A) and another group (*n* = 39) that visited the dentist irregularly (group B). They found that all clinical and radiographic abnormalities were more frequent in group B [dentally unfit (36.4 vs 82.1%), retained roots (9.1 vs 41%), caries (0 vs 25.6%), periodontal disease (35 vs 51.3%) and impacted teeth (0 vs 77%)]. Although statistical tests were not done in this study, the findings indicate that regular dental care is beneficial.

Holbrook *et al*. performed a mirror-and-probe examination of teeth and soft tissue of 100 patients with a cardiac valvular lesion attending a cardiac clinic; six had a history of infective endocarditis.^23^ They found that only 40.5% of the 42 patients with teeth could be regarded as having satisfactory dental health; the remaining patients had either chronic periodontal infection or an abscess, or both. The dental health of edentulous patients was also poor – 53.9% had ill-fitting dentures and 28.9% had diseases of the mouth that could produce bacteraemia.

Smith and Adams published a report on the dental health of 81 at-risk patients attending a cardiology out-patient clinic.^24^ This investigation consisted of a clinical examination and completion of a questionnaire. Edentulous patients were included in the study because of several reports in the literature of edentulous patients suffering from infective endocarditis.^25^-^27^ The criteria used to classify a patient as dentally unfit were provided.

Forty-eight (59.3%) patients were dentally fit; 25 of these patients were edentulous. The dentally unfit group comprised 33 (40.7%) patients, of whom four were edentulous. These workers commented that the prevalence of periodontal disease in their patients was high; however, no figures were given. The articles by Thom and Howe, Holbrook and co-workers, and Smith and Adams, which were based on studies done in the UK, also indicated that a number of edentulous patients were dentally unfit.^22^-^24^

In a study of 38 children with congenital heart disease, dental examination revealed dental caries in 39% of the children.^28^ In another study, 42.4% of 170 children with congenital heart disease had dental caries.^29^ Untreated dental caries were found in 36% of 28 children with previous infective endocarditis or a prosthetic heart valve.^30^

## Conclusion

Our study demonstrates that inadequate attention is paid to the maintenance of good oral health in black patients with severe rheumatic heart disease requiring cardiac surgery. It is very likely that within our healthcare system, the oral health of patients with less severe rheumatic heart disease who are not attending specialist cardiac facilities is also suboptimal. This important aspect of the prevention of infective endocarditis needs greater attention.^16^,^17^
